# Hedgehog signaling induces PD-L1 expression and tumor cell proliferation in gastric cancer

**DOI:** 10.18632/oncotarget.26473

**Published:** 2018-12-21

**Authors:** Jayati Chakrabarti, Loryn Holokai, LiJyun Syu, Nina G. Steele, Julie Chang, Jiang Wang, Syed Ahmed, Andrzej Dlugosz, Yana Zavros

**Affiliations:** ^1^ Department of Pharmacology and Systems Physiology, University of Cincinnati, Cincinnati, OH, USA; ^2^ Department of Molecular Genetics, Biochemistry and Microbiology, University of Cincinnati, OH, USA; ^3^ Department of Dermatology, University of Michigan, Ann Arbor, MI, USA; ^4^ Division of Developmental Biology, University of Michigan, Ann Arbor, MI, USA; ^5^ Department of Biomedical Engineering, University of Cincinnati, Cincinnati, OH, USA; ^6^ Department of Pathology and Laboratory Medicine, University of Cincinnati College of Medicine, Cincinnati, OH, USA; ^7^ Department of Surgery, University of Cincinnati Cancer Institute, Cincinnati, OH, USA; ^8^ Department of Cell and Developmental Biology, University of Michigan, Ann Arbor, MI, USA

**Keywords:** gastric cancer organoids, PD-1, cytotoxic T lymphocytes, dendritic cells

## Abstract

Tumor cells expressing programmed cell death ligand 1 (PD-L1) interact with PD-1 on CD8+ cytotoxic T lymphocytes (CTLs) to inhibit CTL effector function. In gastric cancer, the mechanism regulating PD-L1 is unclear. The Hedgehog (Hh) signaling pathway is reactivated in various cancers including gastric. Here we tested the hypothesis that Hh-induced PD-L1 inactivates effector T cell function and allows gastric cancer cell proliferation. Mouse organoids were generated from tumors of a triple-transgenic mouse model engineered to express an activated GLI2 allele, GLI2A, in Lgr5-expressing stem cells, (mTGOs) or normal mouse stomachs (mGOs). Bone marrow-derived dendritic cells (DCs) were pulsed with conditioned media collected from normal (mGO^CM^) or cancer (mTGO^CM^) organoids. Pulsed DCs and CTLs were then co-cultured with either mGOs or mTGOs in the presence of PD-L1 neutralizing antibody (PD-L1Ab). Human-derived gastric cancer organoids (huTGOs) were used in drug and xenograft assays. Hh/Gli inhibitor, GANT-61 significantly reduced the expression of PD-L1 and tumor cell proliferation both *in vivo* and *in vitro*. PD-L1Ab treatment induced tumor cell apoptosis in mTGO/immune cell co-cultures. GANT-61 treatment sensitized huTGOs to standard-of-care chemotherapeutic drugs both *in vivo* and *in vitro*. Thus, Hh signaling mediates PD-L1 expression in gastric cancer cells and subsequently promotes tumor proliferation.

## INTRODUCTION

The effective diagnosis and treatment of the major gastric cancer risk factor *Helicobacter pylori* (*H. pylori)* has resulted in the decreased incidence of gastric cancer in the United States [1, 2]. However, the incidence of gastric cancer varies throughout the world, with high-risk areas including East Asia (China and Japan), Eastern Europe, and Central and South America [2, 3]. The disease becomes symptomatic in the advanced stages, and the 5-year survival rate for patients diagnosed with this malignancy is only 10%–30% [1, 2, 4]. Given the poor response of gastric cancer to various existing treatment modalities, there is a need for approaches to predict individual therapy responses [1].

Despite the advances of targeted therapy using trastuzumab for HER2-positive gastro-esophageal cancers, anti-VEGFR2 monoclonal antibody ramucirumab and paclitaxel that improve survival, patients with metastatic gastro-esophageal cancer live for less than 2 years [5, 6]. Immune-checkpoint blockade with anti-CTLA4, anti-PD-1 and anti-PD-L1 antibodies has advanced the treatment of many cancers including gastric adenocarcinomas [7]. Programmed death-1 (PD-1) and programmed death ligand-1 (PD-L1) are two- immune-checkpoint molecules for targeted cancer therapy. Tumor cells expressing PD-L1 interact with PD-1 on CD8+ cytotoxic T lymphocytes (CTLs). This interaction inhibits CTL effector function, subsequently leading to immune evasion and cancer cell proliferation [8–10]. PD-L1+ (B7-H1+) gastric cancer stem cells exhibit an increased proliferative capacity [11]. While clinical trials using immune-checkpoint inhibition is proven to be promising for the treatment of gastric cancer, there are no established selection criteria to predict whether a patient will benefit from immunotherapy alone or with combination therapy.

Hedgehog (Hh) signaling plays a crucial role in growth and morphogenesis in a wide variety of tissues during embryonic development [12]. Importantly, the Hh signaling pathway is often overexpressed in various cancers including gastric and pancreatic (reviewed in [13]). Based on the TCGA data, we find that Gli2, Shh, Ptch1, Ptch2, Smo, are altered in 7%, 6%, 10%, 7% and 8% of 258 patients selected for the study, respectively [14]. Importantly, studies suggest that Hh signaling is one of regulatory pathways of PD-L1 expression and that inhibiting Hh signaling may induce lymphocyte anti-tumor activity [15]. Thus, there is interest in targeting the Hh pathway as a potential therapeutic target for the treatment of these cancers. In the current study, we sought to investigate the role of Hh signaling as a mediator of PD-L1 expression during gastric tumorigenesis using an *in vivo* mouse model of gastric cancer, *in vitro* mouse-derived gastric cancer organoid/immune cell co-culture, and human-derived gastric cancer organoid drug assays.

## RESULTS

### Inhibition of Hh signaling results in a decreased PD-L1 expression that correlates with loss of tumor formation in *iLgr5;GLI2A* mice

**Figure 1:**
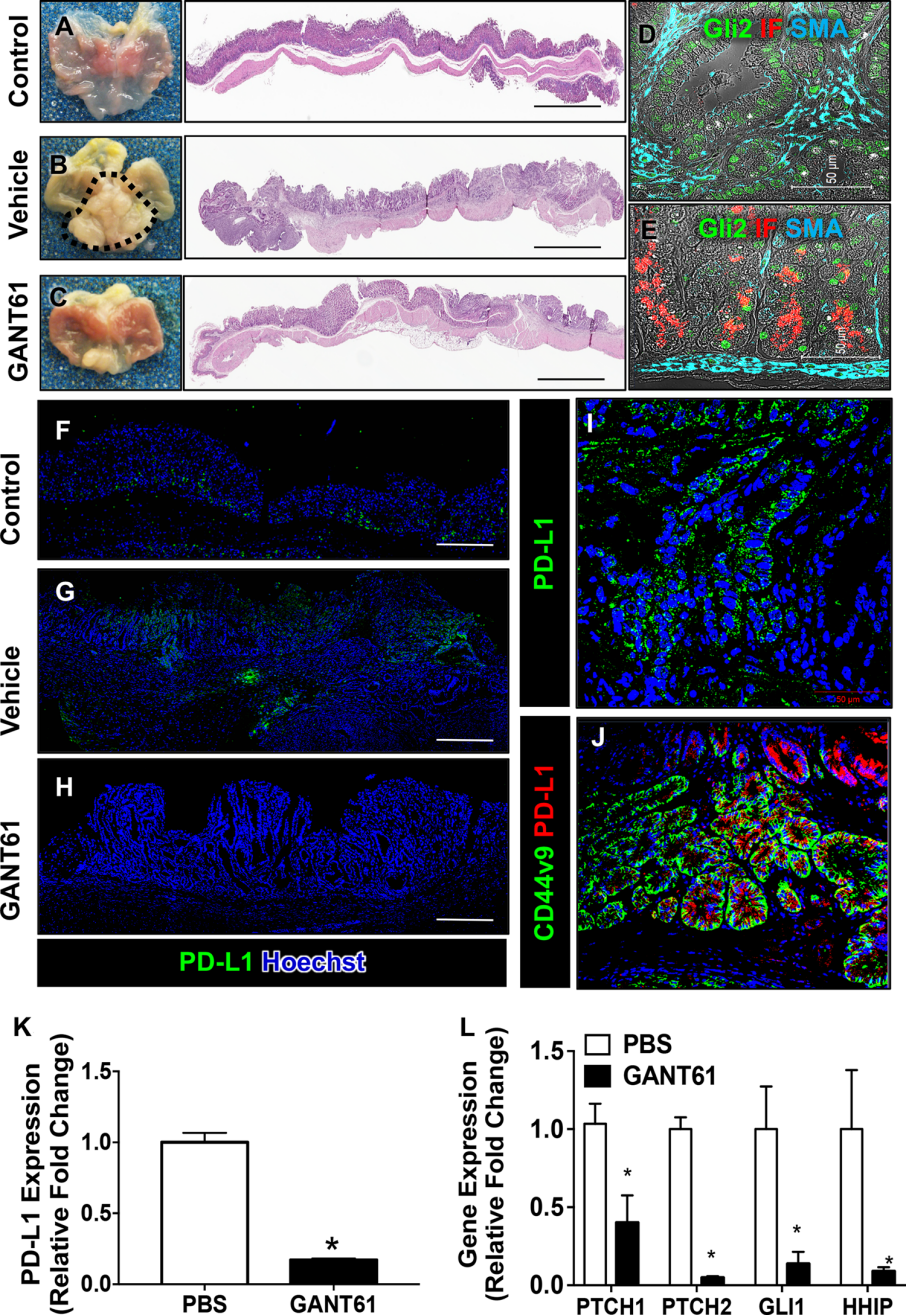
Histological changes and PD-L1 expression in *iLgr5;GLI2A* mice treated with GANT61 H&E staining of sections collected from (**A**) control, (**B**) vehicle treated, and (**C**) GANT61 treated *iLgr5;GLI2A* mice. Immunofluorescence staining for Gli2 (green), intrinsic factor (IF, red) and smooth muscle actin (SMA, cyan) from sections collected from vehicle treated *iLgr5;GLI2A* mice in (**D**) fundus, and (**E**) antral tumor stomach regions. Immunofluorescence staining for PD-L1 expression (green) from sections collected from (**F**) control, (**G**) vehicle treated, and (**H**) GANT61 treated *iLgr5;GLI2A* mice. (**I**) Shows a higher magnification of (**G**). (**J**) Immunofluorescence of CD44v9 (green) and PD-L1 (red) in tumor tissue. Quantitative RT-PCR analysis for expression of (**K**) PD-L1 and (**L**) the downstream Hh canonical signaling genes, *PTCH1*, *PTCH2*, *GLI1* and *HHIP*. ^*^P < 0.05 compared to PBS/vehicle control group, *n* = 5 per group.

To identify whether there was a correlation between induced Hh signaling within the gastric epithelium and induction of PD-L1 expression *in vivo*, expression of PD-L1 was measured in *iLgr5;GLI2A* mice treated with Hh/Gli inhibitor GANT61 (Figure 1). As documented in the original report, activation of GLI2A in Lgr5+ gastric stem cells led to the rapid development of gastric tumors in the antrum after 3 weeks of doxycycline and vehicle treatment (Figure 1B) compared to control treated mice (Figure 1A) [16]. Unlike vehicle treated mice (Figure 1B), *iLgr5;GLI2A* GANT61 blocked the development of adenocarcinoma (Figure 1C). In *iLgr5;GLI2A* mice, within the tumor region Gli 2 (green) was clearly expressed (Figure 1D). Although Gli2 was highly expressed within the IF-positive chief cells of the corpus/fundus of *iLgr5;GLI2A* mice, tumors did not develop in this region of the stomach (Figure 1E). Consistent with studies by Leushacke *et al*. [17], these data would indeed show the induction of Gli2 in Lgr5+ cells within a subset of chief cells in the stomach. However, overexpression of Gli2 in the chief cells does not contribute to tumorigenesis in this region of the stomach, but rather may be important in epithelial regeneration in response to injury as demonstrated by our group [18, 19].

Immunofluorescence staining for PD-L1 revealed increased expression within gastric adenocarcinoma of vehicle treated *iLgr5;GLI2A* mice (Figure 1G, 1I) compared to controls (Figure 1F). PD-L1 expression localized within metaplastic cells that were positive for gastric cancer marker CD44v9 (Figure 1J). The expression of PD-L1 was significantly decreased in the stomachs of mice treated with GANT61 (Figure 1H), a response that was confirmed by qRT-PCR (Figure 1K).

In canonical Hh signaling, Hh ligands bind to the receptor Patched (PTCH) that leads to the activation of key pathway activator Smoothened (Smo), followed by activation of the GLI transcription factors. As a consequence of Smo activation, transcription of Gli target genes including *Gli1*, *PTCH* and *HHIP* are upregulated when the signal is triggered [20]. Decreased PD-L1 expression in response to GANT61 correlated with decreased expression of Hh canonical signaling genes, *PTCH1*, *PTCH2*, *GLI1* and *HHIP* (Figure 1L).

**Figure 2:**
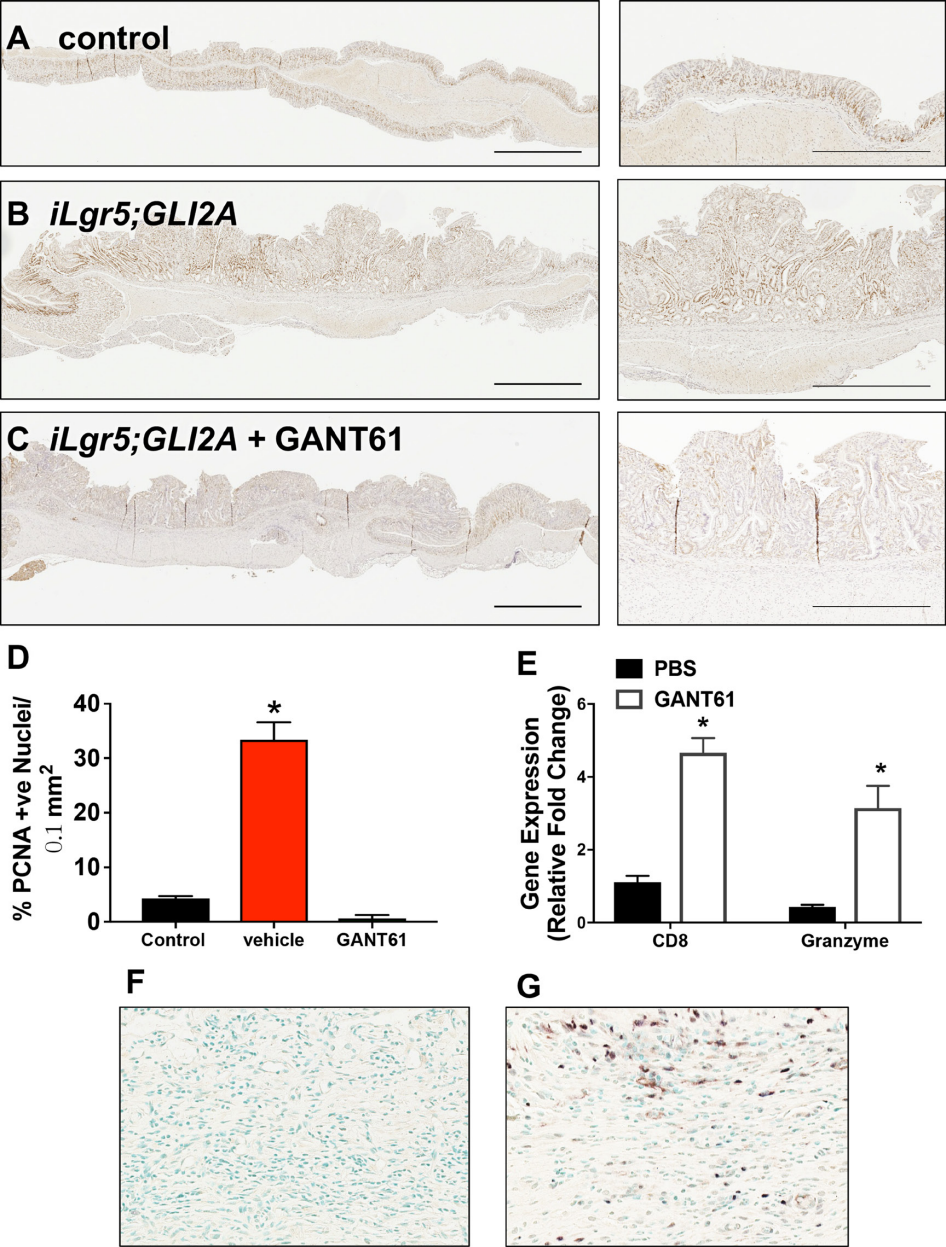
Changes in epithelial and tumor cell proliferation in *iLgr5;GLI2A* mice treated with GANT61 Immunohistochemical staining for PCNA expression (brown nuclei) of sections collected from (**A**) control, (**B**) vehicle treated, and (**C**) GANT61 treated *iLgr5;GLI2A* mice. (**D**) Percentage of PCNA positive nuclei per 0.1 mm^2^ section was quantified. (**E**) Quantitative RT-PCR for the expression of CD8 and granzymeB in tissue collected from vehicle or GANT61 treated mice. ^*^P < 0.05 compared to control group, *n* = 5 per group. Immunohistochemistry for the expression of CD8+ infiltrating cells in tissue collected from (**F**) vehicle or (**G**) GANT61 treated mice.

Immunohistochemical staining for proliferative marker proliferating cell nuclear antigen (PCNA) revealed increased proliferating cells within the tumors of *iLgr5;GLI2A* mice (Figure 2B, 2D) compared to control animals (Figure 2A, 2D). *ILgr5;GLI2A* mice treated with GANT61 had reduced proliferation wtihin the gastric epithelium (Figure 2C, 2D). Immunohistochemical staining showed increased CD8+ cytotoxic T cells infiltrating within GANT61-treated tumors (Figure 2G) compared to control stomachs (Figure 2F). In support of the immunohistochemistry, qRT-PCR data also demonstrated higher expression of CD8 as well as Granzyme B in GANT61 treated mice (Figure 2E). Collectively, these data confirm the previous report demonstrating that activation of GLI2A in Lgr5+ gastric stem cells leads to the development of gastric adenocarcinoma [16]. In addition, with the development of gastric cancer in response to Hh signaling is the robust expression of PD-L1 within tumor tissue.

### Inhibition of Hh signaling results in decreased PD-L1 expression and tumor cell proliferation in *iLgr5;GLI2A* mouse-derived gastric cancer organoids

**Figure 3:**
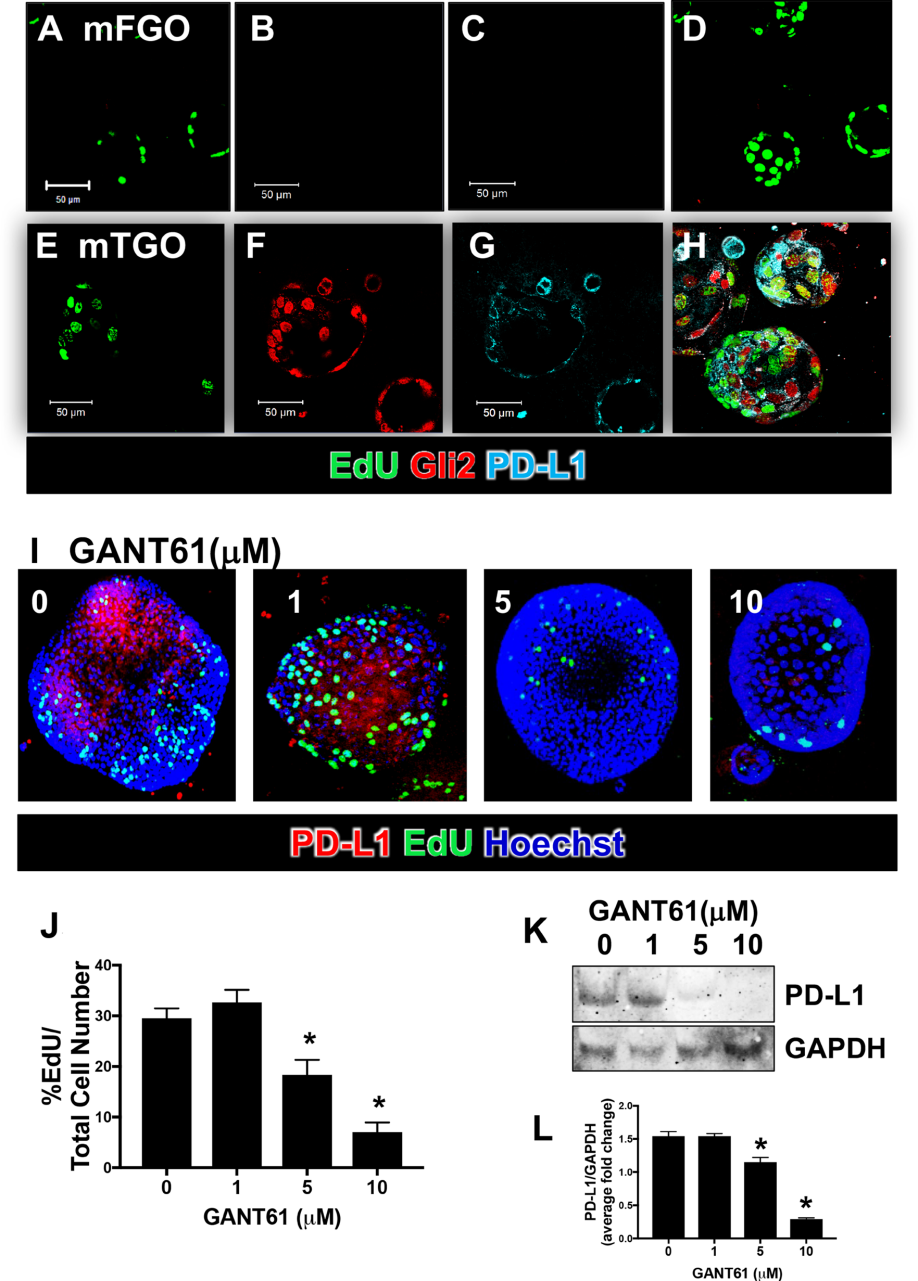
Gli2 and PD-L1 expression in gastric cancer organoids derived from *iLgr5;GLI2A* mice Immunofluorescence staining for proliferating EdU (green), Gli2 (red) and PD-L1 (cyan) expression in organoids derived from control normal (mFGO, **A–D**) and tumor *iLgr5;GLI2A*-derived (mTGO, **E–H**) mouse tissues. (**I**) Immunofluorescence images of PD-L1 (red) and EdU (green) expression in mTGOs, treated with 0, 1, 5, or 10 μM GANT61. (**J**) Quantification of % EdU positive cells/total cells in GANT61 treated mTGOs. ^*^P < 0.05 compared to 0 μM GANT61, *n* = 4 per group. (**K**) Western blot analysis for the expression of PD-L1 using whole cell lysates collected from mTGOs, treated with 0, 1, 5, or 10 μM GANT61. (**L**) Quantification of western blots expressed as the PD-L1/GAPDH average fold change relative to 0 μM GANT61. ^*^P < 0.05 compared to 0 μM GANT61, *n* = 4 per group.

To further identify the role of Hh signaling in the regulation of PD-L1 within gastric cancer cells, we used an organoid model derived from the tumor tissue of *iLgr5;GLI2A* mice (mTGOs). Compared to organoids derived from the stomachs of normal control animals (mFGOs) (Figure 3A–3D), mTGOs were not only significantly more proliferative (mFGO 3.17 ± 1.35% EdU/Total Cell Number, mTGO 43.9 ± 10.7% EdU/Total Cell Number), but also expressed increased Gli2 and PD-L1 expression (Figure 3E–3H). Tumor-derived mTGOs treated with GANT61 showed decreased expression of not only PD-L1 (Figure 3I), but also proliferating EdU-positive cells (Figure 3I, 3J) at concentration of 5 and 10 μM of the inhibitor compared to the vehicle control (0 μM). Western blots showed significantly reduced expression of PD-L1 in response to 5 and 10 μM GANT61 treated mTGOs (Figure 3K, 3L). Our *in vitro* data support the role of Hh signaling as a regulator of cancer cell proliferation and PD-L1 expression.

### Secreted tumor antigen induces PD-1 expression on cytotoxic T Cells (CTLs) and protects cancer organoids from CTL-induced apoptosis

**Figure 4:**
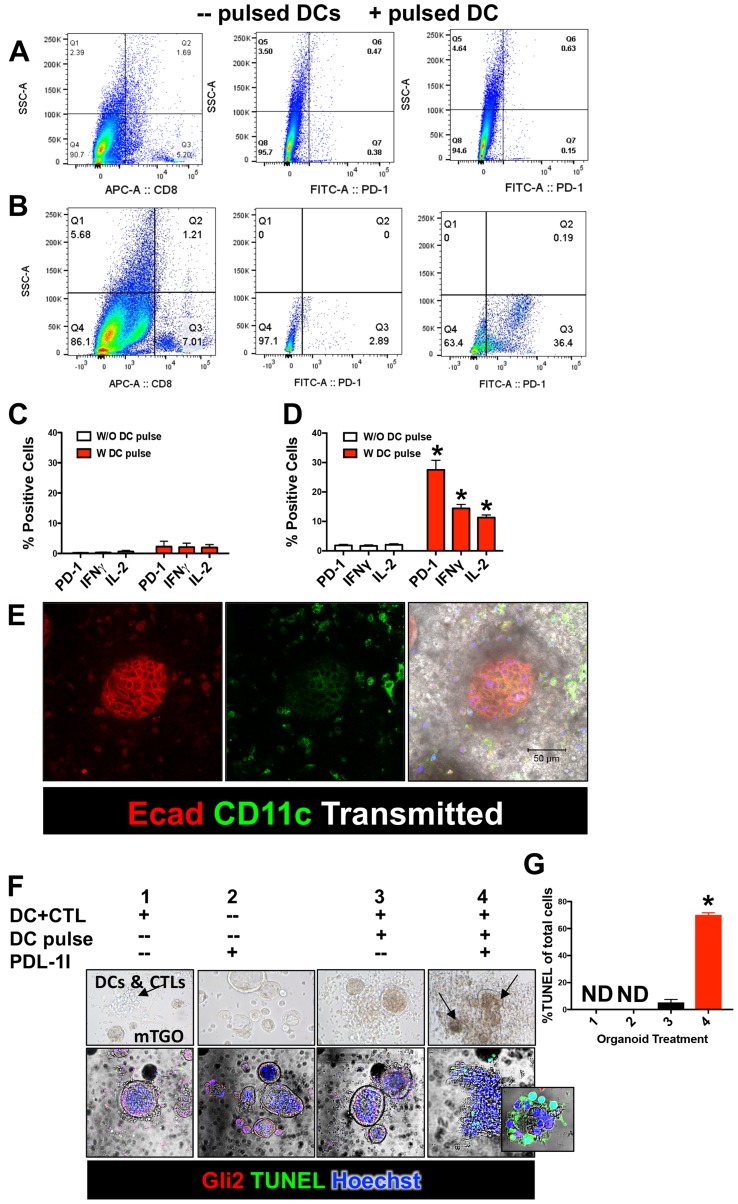
Secreted tumor antigen induces PD-1 expression in cytotoxic T lymphocytes (CTLs) and induce mTGO apoptosis when treated with PD-L1I Flow cytometric histograms showing the expression of CD8 and PD-1 on CTLs co-cultured with DCs pulsed (+pulsed DC) with either (**A**) mGOs^CM^ or, (**B**) mTGOs^CM^. The middle and right panels are sub-gated to the CD8+ cells from the left panels. Quantification of PD-1, IFNγ and IL-2-expressing CTLs co-cultured with DCs pulsed with (**C**) mFGOs^CM,^, or (**D**) mTGO^CM^. ^*^P < 0.05 compared to CTLs exposed to unpulsed DCs, *n* = 4 individual organoid/immune cell co-cultures. (**E**) Representative Immunofluorescence image of mTGO/immune cell co-culture stained for the expression of CD11c (green) and E cadherin (Ecad, red). (**F**) TUNEL stain of organoid/immune cell co-cultures of: 1) mTGOs/DCs/CTLs, 2) mTGOs alone with PD-L1I, 3) mTGOs/pulsed DCs and CTLs, and 4) mTGOs/pulsed DCs/CTLs culture treated with PD-L1I. (**G**) Quantification of apoptotic cells in co-culture conditions 1–4. ^*^P < 0.05 compared to condition 1, *n* = 4 individual organoid/immune cell co-cultures.

To begin to elucidate the role of Hh-induced PD-L1 expression in cancer cell immune evasion, we next developed an autologous culture of gastric cancer organoids, dendritic cells (DCs) and CTLs. Gastric organoids were generated from normal mouse stomachs (mFGO) or *iLgr5;GLI2A* (mTGO) mice [16]. Conditioned media was collected from cultured mFGO^CM^ or mTGO^CM^. DCs were pulsed with either mGO^CM^ or mTGO^CM^ for 24 hours and co-cultured with isolated CTLs for 24 hours. Flow cytometric analysis demonstrated increased expression of PD-1, IFNγ and IL-2 in CTLs co-cultured with DCs pulsed with mTGO^CM^ (Figure 4A, 4D). However, PD-1, IFNγ and IL-2-expressing CTLs co-cultured with DCs pulsed with mFGO^CM^ was not significantly different from expression of CTLs co-cultured with unpulsed DC (Figure 4B, 4C). We then co cultured mGOs or mTGOs with CTLS and DCs, either unpulsed or pulsed with mGO^CM^ /mTGO^CM^ for 48 hrs.

Pulsed DCs and TCs were then co-cultured with mFGOs or mTGOs with and without PD-L1 inhibitor (PD-L1I) and apoptosis measured by TUNEL assay (Figure 4E). Organoid/immune cell co-cultures of: 1) mTGOs co-cultured with DCs and CTLs, 2) mTGOs alone with PD-L1I, 3) mTGOs co-cultured with pulsed DCs and CTLs, and 4) mTGOs co-cultured with pulsed DCs and CTLs treated with PDL1I were performed and apoptosis measured (Figure 4F, 4G). Figure 4F demonstrates the migration of immune cells surrounding the mTGOs in co-culture in response to pulsed DC and CTLs. Representative time-lapse video is shown in Supplementary Video 1. Interestingly, when co-cultures were pretreated with PD-L1 inhibitor there was a significant induction in organoid apoptosis in the wells co-cultured with pulsed DCs (Figure 4F, 4G). Representative time-lapse video is shown in Supplementary Video 2. These data suggest that the inhibition of cancer cell PD-L1 results in cytotoxic T cell-induced tumor apoptosis. Matured DCs were characterized by flow cytometric analysis using specific markers like, CD40, CD80, CD86, CD11c and I-AB (data not shown).

### Inhibition of Hh signaling sensitizes human-derived gastric cancer organoids (huTGOs) to chemotherapy *in vivo* and *in vitro*

**Figure 5:**
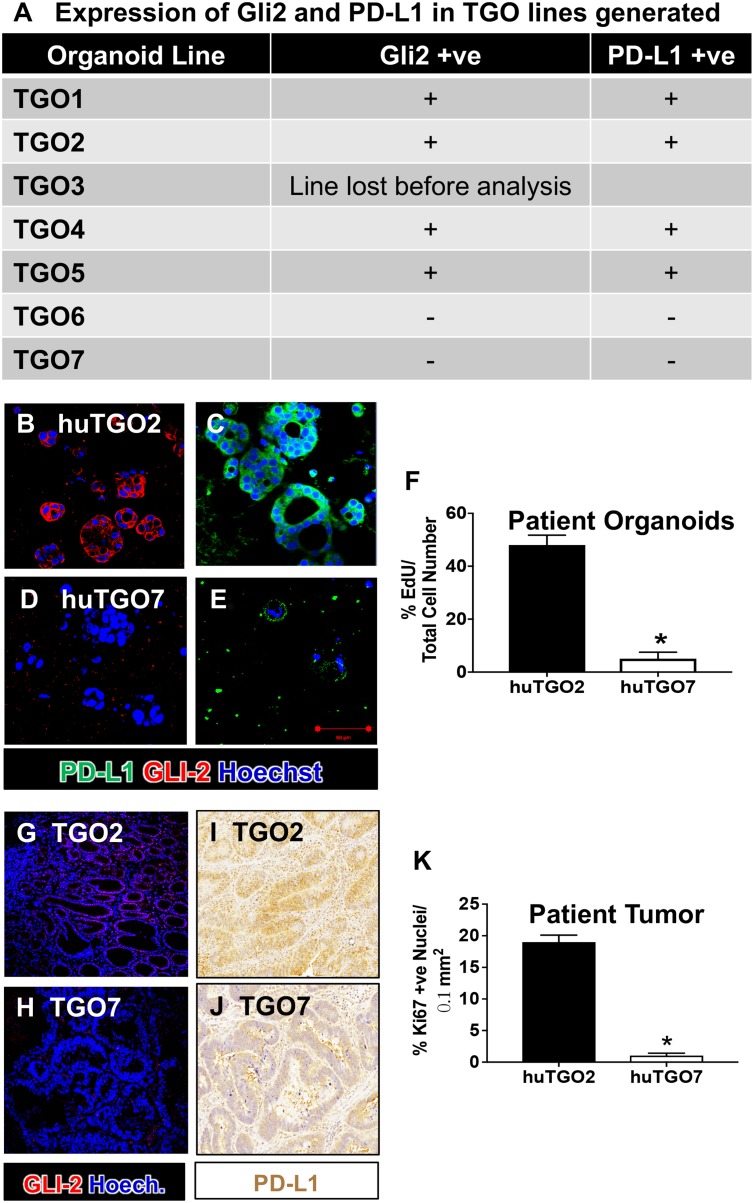
Expression of Gli2 and PD-L1 in huTGO lines and patient tumor tissue (**A**) Expression of Gli2 and PD-L1 in TGO lines generated. Immunofluorescence images for the expression of PD-L1 (green) and Gli2 (red) in (**B**, **C**) huTGO2, and (**D**, **E**) huTGO7. (**F**) Quantification of proliferating cells within huTGO2 and huTGO7 organoid lines. ^*^P < 0.05 compared to huTGO2, *n* = 4 individual organoids. Immunofluorescence staining for Gli2 (red) in patient tumor tissue from which (**G**) huTGO2 and (**H**) huTGO7 were derived. Immunohistochemistry for the expression of PD-L1 in patient tumor tissue from which (**I**) huTGO2 and (**J**) huTGO7 were derived. (**K**) Proliferation in patient tumor tissue from which huTGO2 and huTGO7 were derived. ^*^P < 0.05 compared to tumor tissue from patient from which huTGO2 was derived, *n* = 6 individual high power fields.

The human relevance of Hh signaling in gastric cancer cell viability was addressed using a human-derived gastric cancer organoid model previously reported by our group [21]. We generated human-derived gastric cancer organoids (huTGO) from resected tumors obtained from 7 different patients. Figure 5A reports the Gli2 and PD-L1 expression of all lines based on immunofluorescence staining. Based on the immunofluorescence data, we chose 1 organoid line expressing Gli2 and PD-L1 (huTGO2) (Figure 5B, 5C), and a line that did not express either Gli2 or PD-L1 (huTGO7) (Figure 5D, 5E) based on the robustness of the cultures. There was a significant difference in proliferation between these two organoid line (Figure 5F). Gli2 (Figure 5G, 5H) and PD-L1 (Figure 5I, 5J) expression, and proliferation (Figure 5K) within the patient's native tumor tissue was reflected in the organoid cultures.

**Figure 6:**
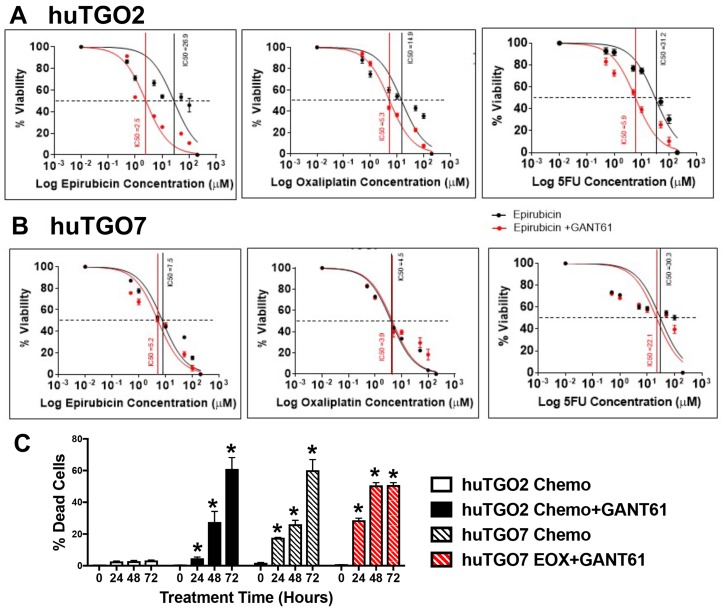
Response of human-derived gastric cancer organoids (huTGO2 and huTGO7) to standard-of-care chemotherapeutic drugs and Hh/Gli inhibitor GANT61 Dose responses epirubicin, oxaliplatin, and 5-Flurouracil (5FU) alone (black curve) or in combination with Hh inhibitor GANT61 (red curve) using (**A**) huTGO2 or (**B**) huTGO7 organoid line. (**C**) Quantification of organoid cell death in response to combination chemotherapy (epirubicin, oxaliplatin and 5FU) alone or with GANT61 using huTGO2 or huTGO7 lines over a 72 hour treatment. ^*^P < 0.05 compared to 0 hour treatment, *n* = 3 individual organoid assays.

**Figure 7:**
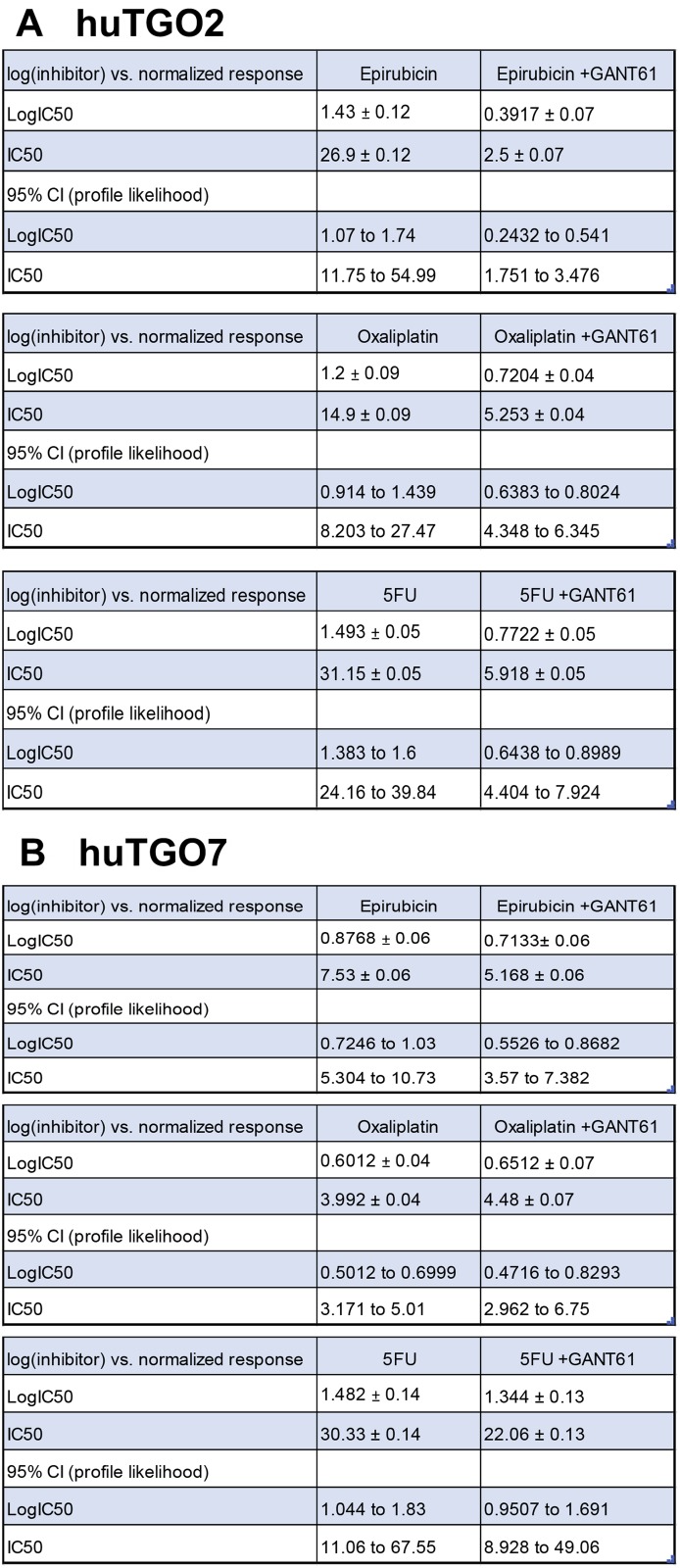
Reported IC50 and 95% CI for doses in response to chemotherapy treatment with GANT61 IC50 and 95% CI for in response to chemotherapeutic drugs alone or in combination with GANT61 using (**A**) huTGO2 or (**B**) huTGO7 lines.

To assess the role of Hh signaling in chemoresistance, huTGOs were treated with standard-of-care chemotherapeutic drugs epirubicin, oxaliplatin and 5-FU alone or in combination with Hh/Gli inhibitor GANT61. Treatment of huTGO2 with GANT61 significantly sensitized the organoids to chemotherapy, as indicated by a shift in the IC50 (Figure 6A, Figure 7A), and induced a significant cell death (Figure 6C). In contrast to huTGO2, treatment of huTGO7 with chemotherapy induced a significant induction in cell death (Figure 6C). GANT61 treatment of huTGO7 did not alter the response to chemotherapy (Figure 6B, 6C, and Figure 7B).

**Figure 8:**
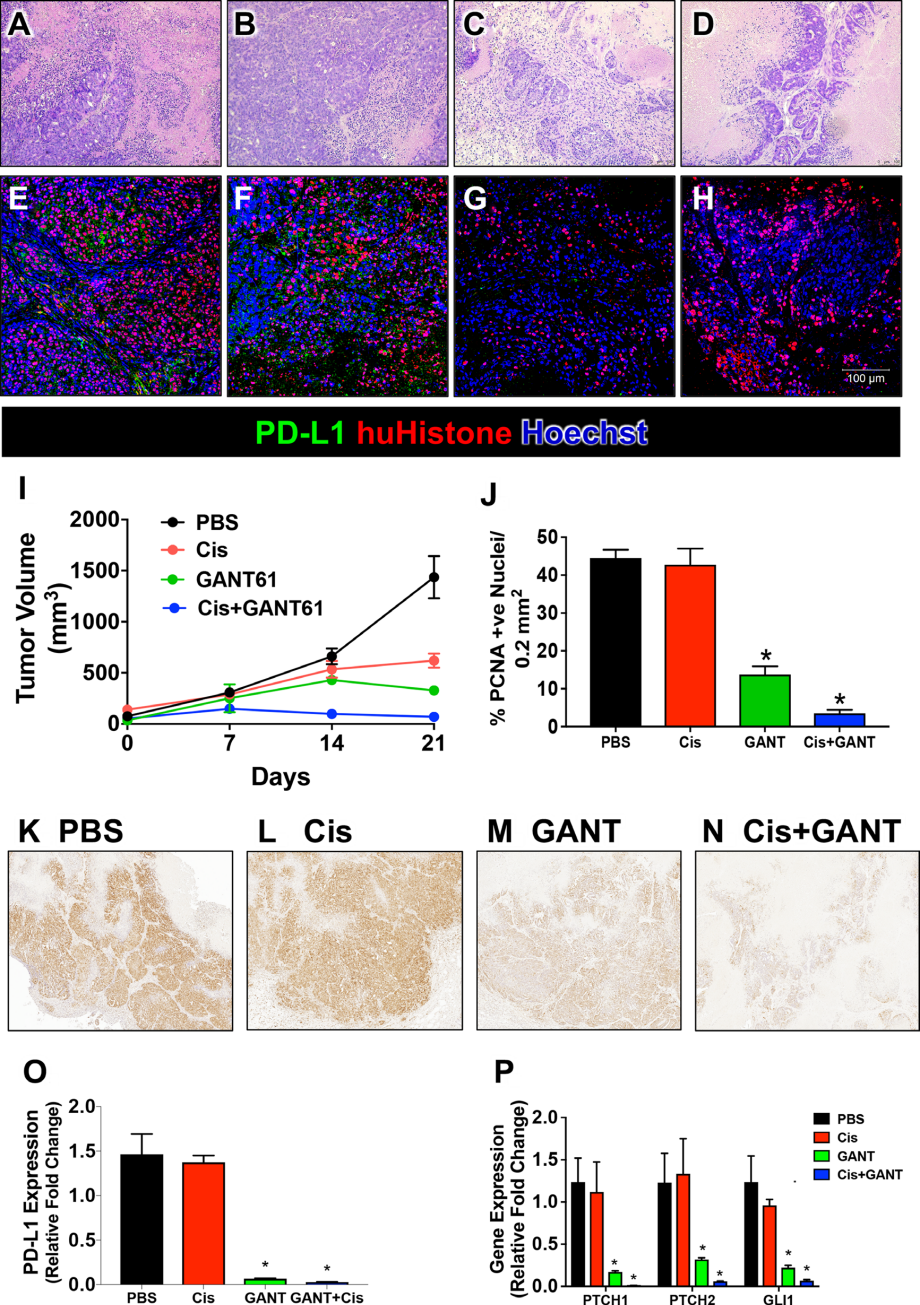
Xenograft mouse model developed using human-derived gastric cancer organoids (huTGO2) H&E and immunofluorescence staining for PD-l1 (green and human-specific histone (huHistone, red) of sections collected from xenografted mice treated with either (**A**, **E**) vehicle, (**B**, **F**) Cisplatin (Cis), (**C**, **G**) GANT61 or (**D**, **H**) Cis+ GANT61. (**I**) Tumor volume (mm^3^) measured in huTGO2 xenograft model. (**J**) Percentage of PCNA positive nuclei per 0.1 mm^2^ section. Representative immunohistochemical stains of sections collected from xenografted mice treated with either (**K**) vehicle, (**L**) Cis, (**M**) GANT61 or (**N**) Cis+ GANT61. Quantitative RT PCR measuring the expression of (**O**) PD-L1 and (**P**) canonical Hh signaling genes, *PTCH1*, *PTCH2* and *GLI1*. ^*^P < 0.05 compared to vehicle/PBS group, *n* = 5 per group.

The huTGO2 line was used in a xenograft model. Mice were then treated with either vehicle (PBS), cisplatin (Cis), GANT61 or Cis+GANT61. Histological evaluation revealed the development of adenocarcinoma in mice xenografted with huTGO2 (Figure 8A). Immunofluorescence using an antibody specific for human histone confirmed the engraftment of human-derived cells (Figure 8E). Histology was reflected in tumor volume (Figure 8I). Compared to tumor growth that was observed in vehicle treated mice (Figure 8A, 8E, 8I), Cis (Figure 8B, 8F, 8I) and GANT61 (Figure 8C, 8G, 8I) alone treated mice had significantly decreased tumor volume 21 days post-xenograft. Importantly, combination treatment of Cis+GANT61 was the most effective at inhibiting tumor growth over time (Figure 8D, 8H, 8I). GANT61 (Figure 8J, 8M) or Cis+GANT61 combined (Figure 8J, 8N) correlated with decreased tumor cell proliferation of tumor growth. There was no statistically significant difference between the vehicle (Figure 8J, 8K) and Cis alone (Figure 8J, 8L) treated groups.

We investigated the changes in PD-L1 expression in the treated xenograft mouse model transplanted with huTGO2. The development of adenocarcinoma in vehicle treated mice correlated with expression of PD-L1 (Figure 8E, 8O). While, Cis alone (Figure 8F, 8O) treatment had no effect on PD-L1 expression, GANT61 alone (Figure 8G, 8O) or in combination with Cis (Figure 8H, 8O) resulted in decreased PD-L1 expression within tumor tissue collected from the xenografts (Figure 8H, 8O). Decreased PD-L1 expression in response to GANT61 or GANT61+Cis correlated with decreased expression of Hh canonical signaling genes, *PTCH1*, *PTCH2*, and *GLI1* (Figure 8P). Collectively, these data suggest that in gastric cancer cells expressing Gli2, inhibition of Hh signaling sensitizes resistance to chemotherapy.

## DISCUSSION

Our data show that Hh signaling plays a role in mediating PD-L1 expression during gastric cancer development. In support of our findings, studies in pancreatic ductal adenocarcinoma cell lines revealed that Hh signaling induced PD-L1 expression under hypoxic conditions [15]. Evidence in the literature also demonstrates that the induction of PD-L1 is largely mediated by proinflammatory cytokines, of which IFNγ is the most potent [22]. The intrinsic induction of PD-L1 is mediated via several oncogenic and transcriptional pathways. For example, in glioblastoma PD-L1 upregulation correlates with loss of PTEN [23]. In addition, activation of PI3K and mTOR pathway, or the epidermal growth factor receptor/mitogen-activated protein kinase pathway correlates with the overexpression of PD-L1 [24, 25]. In a co-culture of Panc-1 cells with lymphocytes, blockade of PD-L1, with either anti-PD-L1 antibody or Hh inhibitor cyclopamine, resulted in induced lymphocyte anti-tumor activity [15]. We extend these findings both *in vivo* using a mouse model of gastric cancer, and *in vitro* using a gastric cancer organoid culture. *In vivo*, in a mouse model of gastric cancer that was developed by overexpressing GLI2A in Lgr5+ gastric stem cells (*iLgr5;GLI2A* mice) [16], GANT61 inhibition of adenocarcinoma correlated with two effects that included reduced 1) PD-L1 expression, and 2) tumor proliferation that resulted in increased CD8+ cytotoxic T lymphocytes. These data suggest that GANT61 partially mediates tumor suppression via reduced PD-L1 expression. This is evident from the findings in Figures 6 and 8, whereby in Figure 6 there is an absence of CTL and thus we may consider that organoid death is due to loss of proliferation. In contrast, in Figure 8 *in vivo* the effect of GANT61 on tumor growth may also be mediated by inhibition of PD-L1. Moroever, *in vitro* using gastric cancer organoids derived from *iLgr5; GLI2A* mice, GANT61 similarly reduced PD-L1 expression and cancer cell proliferation. Interestingly, *in vivo* GANT61 inhibition of Gli2-induced PD-L1 by tumor cells, resulted in increased CTL numbers within the tumor tissue. The PD-1/PD-L1 pathway is involved in controlling the proliferation and cytokine production of T cells [26]. Thus, a plausible explanation may be that the observation is attributed to increased proliferation of resident CTLs within the tumor tissue rather than infiltration of CD8+ cells. Overall, the relationship between PD-L1 expression and prognosis in gastric cancer is still controversial [27]. While some studies show that PD-L1 expression correlates with an adverse prognosis, others show that patients with PD-L1 positive tumor cells have an improved prognosis [28, 29]. Therefore, such information makes it difficult to predict the effectiveness of immune-checkpoint inhibition for the treatment of gastric cancer.

**Figure 9:**
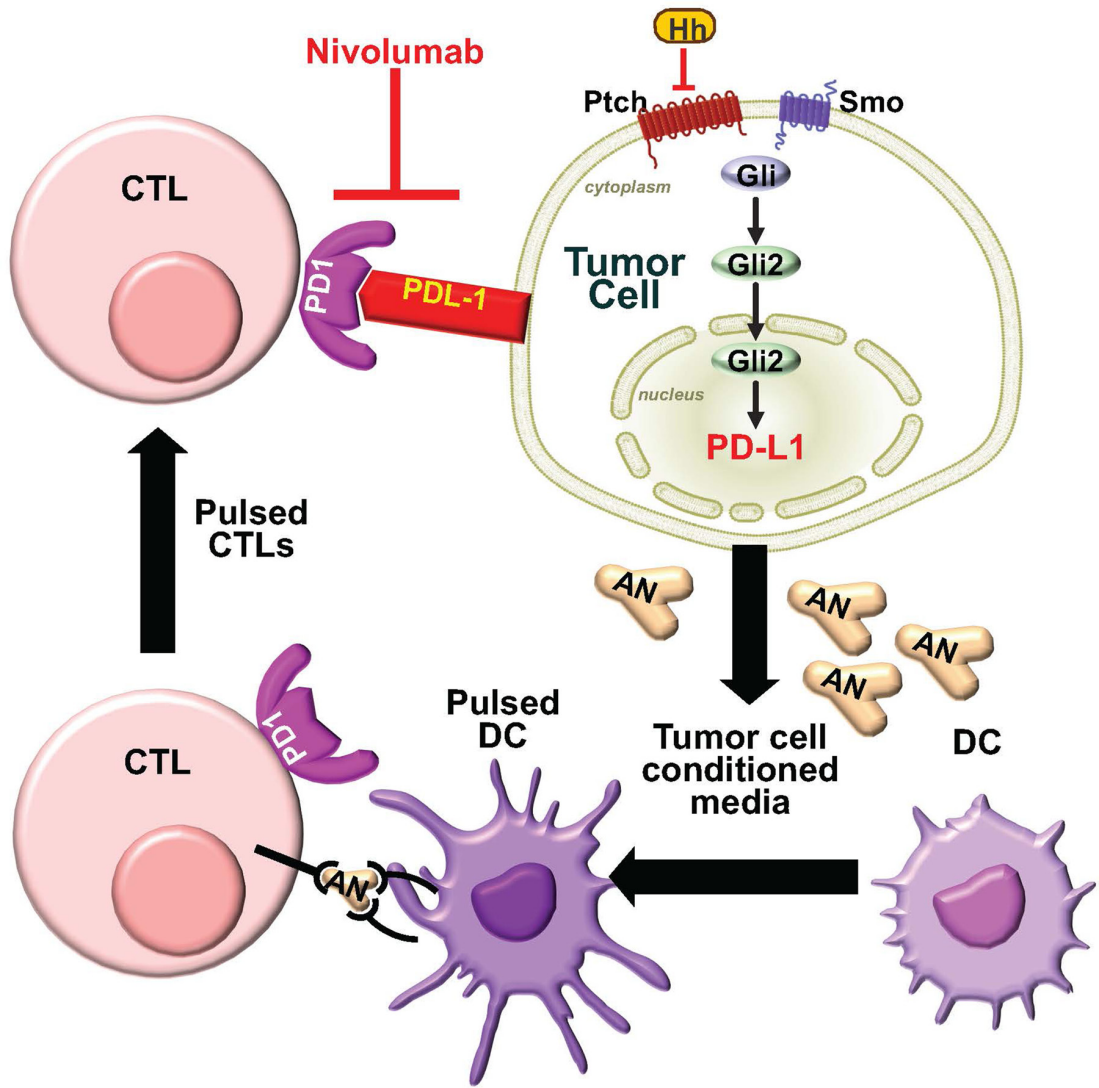
Proposed model for the induction of PD-L1 by Hh signaling We proposed from these studies that Hh signaling induces the expression of PD-L1 on gastric tumor cells. When antigen presenting dendritic cells (DC) are pulsed with conditioned media collected from gastric cancer organoids, containing tumor-specific antigen (AN), and co-cultured with CTLs, there is a significant induction of PD-1 on these lymphocytes. Thus, secreted tumor-specific AN induces PD-1 expression on CTLs. The co-culture of CTLs exposed to tumor-specific AN with gastric cancer organoids resulted in the protection of cancer cells from anti-tumor lymphocyte activity. Immune-checkpoint inhibition using anti-PD-1 inhibitor Nivolumab resulted in cancer organoid death.

Here, we generated an autologous organoid/immune cell co-culture to understand the feasibility for the use of such a model to potentially predict the efficacy of immune-checkpoint inhibition for the treatment of gastric cancer. The first significant observation that we made from this culture was that secreted tumor antigen induced PD-1 expression on CTLs. When antigen presenting dendritic cells were pulsed with conditioned media collected from gastric cancer mTGOs and co-cultured with CTLs, we observed a significant induction of PD-1 on these lymphocytes. Second, the co-culture of CTLs exposed to tumor-specific antigen with mTGOs resulted in the protection of cancer cells from anti-tumor lymphocyte activity. Immune-checkpoint inhibition resulted in mTGO death. Tumor-associated antigens are antigens expressed on tumor cells that can elicit an immune response within the tumor microenvironment. In order to induce tumor-specific T cells, immunogenic peptides derived from tumor-associated antigens must be presented to T cells by efficient professional antigen-presenting cells, such as dendritic cells, which are able to activate naïve and memory T cells [30, 31]. Under ideal circumstances, immunogenic peptides derived from tumor-associated antigens presented on the surface of tumor cells with MHC-I activate cytotoxic T cells and subsequently results in the lysis of tumor cells [31, 32]. As our data suggests, the tumor-associated antigen secreted within the conditioned media of mTGOs induced PD-1 on CTLs leading to immune evasion by the cancer cells. The overall goal of the study was to develop a culture system to test whether inhibition of Gli2-PDL1 would lead to tumor cell apoptosis. To test this hypothesis, the development of an autologous model was necessary, in which tumor antigens were used to cross-present to CTLs. The result of these studies showed that PD-1/PD-L1 inhibition reversed the tolerance induced by Gli2. The mouse organoid/immune cell co-culture may be a promising preclinical model. Future studies using patient-derived gastric cancer organoids and autologous patient-derived immune cells in such a co-culture may be beneficial to testing immunotherapy for each individual patient. Importantly, we report that Hh signaling contributes to the induction of PD-L1 expression in gastric cancer, suggesting that combinatorial drug therapy using Hh signaling and immune checkpoint inhibition may be suitable for candidate patients (Figure 9).

To investigate the role of Hedgehog signaling in the regulation of PD-L1 expression, we used *in vivo* and *in vitro* organoid cultures derived from gastric cancer patients. Human-derived gastric cancer organoids (huTGOs) that expressed Gli2, not only highly expressed PD-L1, but were also chemoresistant to standard-of-care drugs. Combinatorial drug treatment of huTGOs with both Hh inhibitor GANT61 and chemotherapeutic drugs resulted in decreased cancer cell proliferation and induced cell death both *in vitro* and *in vivo*. These data suggest that activation of the Hh signaling pathway renders cancer cells resistant to immunotherapy. In support of our findings, Activation of Hh pathway components including Smo, PTCH and Gli1 in ovarian epithelial cells resulted in cisplatin resistance [33]. In a separate experiment, co-administration of IPI-926, a drug that depletes tumor-associated stromal tissue by inhibiting the Hh signaling pathway in a xenograft model of pancreatic ductal adenocarcinoma enhanced the delivery of gemcitabine, decreased tumor cell proliferation and disease progression [34]. Treating the patient's organoids together with the patient from which the cultures were derived will potentially test the advantage of this approach to predict the best therapeutic option. Overall, our data presents new avenues for improving the efficacy of therapeutics in patients with gastric cancer.

## MATERIALS AND METHODS

### Mice

All mouse studies were approved by the University of Cincinnati Institutional Animal Care and Use Committee (IACUC) that maintains an American Association of Assessment and Accreditation of Laboratory Animal Care (AAALAC) facility. We used a triple-transgenic model engineered to express an activated GLI2 allele, *GLI2A*, in Lgr5- expressing stem cells and their progeny in adult mice when treated with tamoxifen followed by doxycycline [16]. The triple-transgenic *Lgr5-EGFP-IRES-CreERT2; R26-LSL-rtTA-IRES-EGFP; tetO-GLI2A* mouse is abbreviated as *iLgr5; GLI2A*. ILgr5; GLI2A mice were treated with either vehicle (PBS, i.p., every alternate day), or GANT61 (50 mg/kg body weight, i.p., every alternate day; Stem Cell Technologies, 73692), after the completion of oral doxycycline treatment for 3 weeks. Stomach tissues were collected and analyzed for histology, immunostaining and qRT PCR.

### Generation of mouse and human organoids

Mouse-derived gastric cancer organoids (*iLgr5; GLI2A* mTGOs) were generated from the stomachs using a previously published protocol [21]. Briefly, tumor tissue was collected in cold Dulbecco's phosphate buffered saline (DPBS) without calcium or magnesium (w/o Ca/Mg) and with 1% penicillin/streptomycin, 1% kanamycin and Amphotericin B (0.25 mg/ml)/Gentamicin (10 mg/ml). The tissues were minced and incubated in 10 mL pre-warmed EDTA stripping buffer (HBSS with 20% FCS, 1% pen/step, HEPES and EDTA) at 37°C in a rotating shaker, for 10 min followed by another 5 min with fresh EDTA buffer. Tissue was washed with Dulbecco's Modified Eagle's medium (DMEM) supplementated with 1% penicillin/ streptomycin, 1% kanamycin and Amphotericin B (0.25 mg/ml)/Gentamicin (10 mg/ml) twice and incubated with 10 mL pre-warmed collagenase A-hyaluronic acid buffer at 37°C in a rotating shaker for 15–30 min. Digested tissue was then filtered through 40 μm filter. Filtered cells were centrifuged at 1200 rpm for 5 min, washed with DPBS and centrifuged at 400xg for 5 min. The supernatant was discarded and the pellet was resuspended in the desired amount of Matrigel™ supplemented with 1% Kanamycin and 1% Penicillin/streptomycin and seeded into culture plates overlaid with gastric organoid media ((DMEM/F12 supplemented with 10 mM HEPES, 1X Glutamax, 1% Pen/Strep, 1X N2, 1X B27, 1 mM N-Acetylcystine, 50 ng/mL EGF, 100 ng/mL Noggin, 10% R-Spondin Conditioned Media, 50% Wnt Conditioned Media, 100 ng/mL FGF10, 10 nM Gastrin, 10 uM Y-27632) and incubated at 37°C CO_2_ incubator. Normal mouse gastric organoids (mGO) were generated from mouse stomach using a previously published protocol [35]. Human tumor tissue was obtained from patients undergoing surgical resection for gastric cancer after consent (IRB protocol number: 2015-5537, University of Cincinnati). Tumor organoids were generated as previously published protocol [21].

### Generation of bone marrow-derived dendritic cells

Bone marrow cells were collected from either control or *iLgr5;GLI2A* mice. The femur and tibia were collected and separated without disturbing the bone marrow. The bone marrow was flushed into a 50 ml tube using a 26G needle and R10 media (RPMI-1640, Fisher Scientific, 10041CV supplemented with, 1% Penicillin/streptomycin, and, 10% FCS), filtered through 40 μm filter, centrifuged at 423g for 5 minutes at 4°C. Cells were resuspended in complete dendritic cell (DC) media (RPMI-1640 supplemented with 50 μM β-mercaptoethanol (ThermoFisher Scientific, #21985023), 1% Penicillin/streptomycin, 10% FCS and, 20 ng/mL rmGMCSF (R&D System, 415-mL-010)) and plated 1 mL of cells/well of a 6 well tissue culture plate. Fresh DC media was added to each well on day 3 of the culture [36]. DCs were cultured for 5 days before pulsed with organoid conditioned media (CM).

### Isolation and cryopreservation of mouse splenocytes and culture of cytotoxic T lymphocytes (CTLs)

Spleens were collected from either control or *iLgr5;GLI2A* mice in cold PBS/2% FBS. Each spleen was placed on top of 40 μm cell strainer and disrupted with a flat plunger tip of a 10 mL syringe. Splenocytes were centrifuged at 300 xg for 10 minutes and resuspend in freezing medium (1 × 10^8^ cells/mL RPMI supplemented with 65% FCS and 10% DMSO), and frozen at –80°C until organoids were ready for co-culture. Frozen splenocytes were quickly thawed in a 37°C water bath in pre-warmed thawing media (RPMI supplemented with 10% FCS, and, 1% Penicillin/streptomycin). The cells were centrifuge at 270 xg for 5 min, CTLs were then isolated using the EasySepTM Mouse CD8+ T cell Enrichment according to manufacturer's protocol (Stemcell Technologies # 19853). CD8+ T cells (CTLs) were resuspended in T-Cell media (RPMI-1640 supplemented with 50 μM β-mercaptoethanol (Thermo Fisher Scientific, #21985023), 1% ITS (1.7 μM Insulin, 68.8 μM Transferrin, 3.9 nM Selenite; ThermoFisher Scientific, #41400045), 1% Penicillin/streptomycin, 10% FCS, 0.25 mg/ml Amphotericin B /10 mg/mL Gentamicin, 50 mg/ml Kanamycin, 30U/mL (6 ng/mL) mouse recombinant interleukin 2 (IL-2, Mitenyl Biotec, 130-094-055) and, 5 ng/mL mouse recombinant interleukin 7 (IL-7, Stemcell Technologies, 78054-1)) and cultured in a 6 well plate at a density of 2 × 10^6^ cells/well for 24 hours before co-culture.

### Organoid/immune cell co-culture

After 5 days of culture, DCs were harvested, resuspended in 750 μL DC media and seeded back to same wells with 100 μL of conditioned media collected from normal/control (mGO^CM^) or *iLgr5;GLI2A* mouse-derived (mTGO^CM^) organoids. DCs were pulsed with conditioned media for 24 hours. On the same day, CTL culture was started. After 24 hours of culture, CTLs were centrifuged at 270 xg for 5 min and resuspend in 750 μL TC media and added to the matured and pulsed DCs. At this stage IL-7 was withdrawn from the wells where conditioned media was added. Pulsed DCs and CTLs were co-cultured for 24 hours.

Suspended or loosely adherent DCs and CTLs were harvested by centrifugation. Adherent DCs were harvested using cell dissociation buffer (1 mL/well, ThermoFisher Scientific, 13151-014). At that time mGOs or mTGOs were harvested in cold PBS, centrifuged at 406 xg for 5 min and resuspended in cold PBS combined with DCs and CTLs. Organoids, DCs and CTLs were centrifuged at 406 xg for 5 min and resuspended in the desired amount of Matrigel™. A 30 μL Matrigel™ bubble was seeded in each well overlaid with gastric organoid media. Experimental groups included: 1) mGOs or mTGOs plus DCs and CTLs, 2) mGOs or mTGOs alone treated with PD-1 inhibitor (270 pg/mL, Nivolumab, Selleckchem, A2002), 3) mGs or mTGOs plus pulsed DCs and CTLs, and 4) mGs or mTGOs plus pulsed DCs and CTLs treated with PD-L1 inhibitor in 8 well chambered coverglass. Organoids were analyzed by TUNEL staining according to the manufacturer's protocol (Thermo Fisher scientific, C10245). A time-lapse video with brightfield images were acquired every 15 min up to 16 hrs using Inverted Zeiss LSM 710 confocal microscope, equipped with 37°C incubator and 5% CO_2_.

### Flow cytometric analysis of CTLs and DCs

CTLs were collected in media from individual wells and added to 15 mL conical tubes. Dendritic cells were dissociated from the wells using Cell Dissociation Buffer (Life Technologies, #13151-014) and added to the same tube. The cells were centrifuged at 300 x g for 5 mins and resuspended in 100 μl 5% BSA in PBS. CD8a (APC, Biolegend, 100711) and PD1 (FITC, Biolegend, 135213) were added (1:100) in the cell suspension. The cells were incubated in the dark at room temperature for 15 mins. Reagent A fixation medium (Invitrogen, #GAS001) was added to the cell suspension for 15 mins at room temperature. 5% BSA in PBS was added to the cells and the suspension was centrifuged at 300g for 5 mins. The cell pellets were resuspended in 100 μl Reagent B permeabilization buffer (Invitrogen, GAS002). Primary antibodies IFN-γ (PE, Biolegend, 505807) and IL-2 (Brilliant Violet 421, Biolegend, 503825) were added (1:100) in the cell suspension and incubated for 20 mins at room temperature in the dark. 5% BSA diluted in PBS was added to the cell suspension and centrifuged at 300g for 5 min. The labeled CTLs were resuspended in 5% BSA in PBS and the activation was analyzed using Canto-III flow cytometer. In a separate series of cultures, DCs were pulsed with mTGO^CM^. The matured DCs were collected and immunostained with CD40 (APC, Biolegend, 124611), CD80 (BV421, Biolegend, 104725), CD86 (PE, Biolegend, 105105), CD11c (BV650, Biolegend, 117339), and I-AB (FITC, Biolegend, 114406). Labeled DCs were then analyzed using Canto-III flow cytometer and FlowJo data analysis program.

### Quantitative RT-PCR (qRT-PCR)

RNA was isolated from either stomach tissue of *iLgr5;GLI2A* mice or xenografted tumor tissue using TRIzol Reagent (Molecular research Center, TR118) according to the manufacturer's instructions. The High Capacity cDNA Reverse Transcription Kit (Applied Biosystems) was used for cDNA synthesis of RNA following the manufacturer's protocol. For each sample 100 ng of RNA was reverse transcribed to yield approximately 2 μg total cDNA that was then used for the real-time PCR. Pre-designed Real-Time PCR assays were purchased for the following genes (Thermo Fisher, Applied Biosystems): Mouse (Mm) or human (Hs)-specific GAPDH (Hs02786624_g1; Mm99999915_g1), PD-L1 (Mm03048248_m1, Hs00204257_m1), PTCH1 (Mm00436026_m1; Hs00181117_m1), PTCH2 (Mm00 436047_m1; Hs00184804_m1), GLI1 (Mm00494654_m1; Hs00171790_m1), HHIP (Mm00469580_m1; Hs0101015_m1), CD8 (Mm01182107_g1), and GranzymeB (Mm0 1322702_m1). PCR amplifications were performed in a total volume of 20 μl, containing 20X TaqMan Expression Assay primers, 2X TaqMan Universal Master Mix (Applied Biosystems, TaqMan^®^ Gene Expression Systems) and cDNA template. Each PCR amplification was performed in duplicate wells in a StepOne™ Real-Time PCR System (Applied Biosystems), using the following conditions: 50°C 2 minutes, 95°C 10 minutes, 95°C 15 seconds (denature) and 60°C 1 minute (anneal/extend) for 40 cycles. Fold change was calculated as: (C_t_–C_t high_) = n_target_, 2^ntarget^/2^nHPRT^ = fold change where C_t_ = threshold cycle. The results were expressed as average fold change in gene expression relative to control with GAPDH used as an internal control according to Livak and Schmittgen [37].

### Western blot

Organoids were lysed in M-PER Mammalian Protein Extraction Reagent (Thermo Scientific, 78501) supplemented with protease inhibitors (Roche, 05 892 970 001) according to the manufacturer's protocol. Cell lysates were resuspended in 40 μl Laemmli Loading Buffer containing β-mercaptoethanol (Bio-Rad Laboratories, 1610730) before western blot analysis. Samples were loaded onto 4–20% Tris-Glycine Gradient Gels (Invitrogen) and run at 80 volts for approximately 3 hours and transferred to nitrocellulose membranes (Whatman Protran, 0.45 μM) at 105 volts for 1.5 hours at 4°C. Membranes were blocked for 1 hour at room temperature using KPL Detector Block Solution (Kirkegaard & Perry Laboratories, Inc., 718300). Membranes were incubated for 16 hours at 4°C with either 1:100 dilution of rat anti-PD-L1 (Novus Biologicals, NBP1-76769), or 1:2000 dilution of mouse anti-GAPDH (Millipore, MAB374) antibodies followed by 1 hour incubation with a 1:1000 dilution anti-mouse, or -rat Alexa Fluor 680 (Invitrogen). Blots were imaged using a scanning densitometer along with analysis software (Odyssey Infrared Imaging Software System) and the ratio of PD-L1/GAPDH was calculated using ImageJ.

### Drug assay in human tumor-derived organoids

Human-derived gastric cancer organoids (huTGOs) were grown in 96 well plates and treated with either epirubicin (Selleckchem, S1223), oxaliplatin (Sigma-aldrich, 9512), or 5-Fluorouracil (Selleckchem, S1209) at concentrations of 0, 0.5, 1, 5, 10, 50, 100 and 200 μM or in combination with 5 μM GANT61 (Stemcell Technologies, 73692) for 48 hours. After 48 hours, organoid proliferation was measured using MTS Assay (Promega, G3580). The absorbance was measured at 495 nm, concentrations were transformed in logarithmic scale, and a best fit nonlinear dose-response curve was plotted using GraphPad Prism (GraphPad Software, San Diego, CA). huTGOs were also grown in 48 well tissue culture plates and treated with either DMSO, or, the combination of epirubicin, oxaliplatin, and 5-FU at their calculated IC50 concentration, or 5 μM GANT61 plus a combination of epirubicin, oxaliplatin, and 5-FU at their calculated EC50 concentration. The organoids were harvested at different time points (0, 24, 48 and 72 hrs), resuspended in 5% BSA in PBS and stained with calcein AM and Ethidium homodimer1 for 30 min, room temperature at dark (ThermoFisher Scientific, L3224). Labeled cells were analyzed using Canto-III flow cytometer. The average ratio of live/dead cells were calculated using FlowJo and plotted as mean ± SEM using GraphPad Prism software.

### Mouse xenograft assay

Xenograft assays were performed by injecting organoids (huTGO2) subcutaneously in the right flank of NSG mice. Tumor dimensions were measured every 7 days. In mice transplanted with gastric cancer-derived organoids, once tumor volume reached 100 cubic millimeters (within 14 days of transplantation), animals were treated with either vehicle (PBS, i.p.), or GANT61 (50 mg/kg in PBS, i.p. every alternate days), or cisplatin (3 mg/kg in saline, i.p. 2 days/week, Selleckchem, S1166), or cisplatin plus GANT61 for 30 days. Tumor height (h), length (l) and width (w) were measured once/week using a caliper. Tumor volume was calculated using a published equation [38].

### Immunofluorescence

Stomach tissues were fixed in 4% paraformaldehyde for 16 hours, paraffin-embedded and sectioned at 5 μm. Tissue slides were deparaffinized and boiled in antigen citrate buffer (Vector Laboratories, H3300) for 10 minutes. Sections were then blocked with 20% donkey serum for 20 minutes, and immunostained with primary antibodies overnight at 4°C, followed by incubation with secondary antibodies for 1 hour. Coverslips were mounted onto slides with Vectashield Mounting Medium (Vector Laboratories, H-1400). For whole mount staining, gastric organoids were fixed in 3.7% formaldehyde for 15 minutes at room temperature, followed by washing in DPBS. Organoids were permeabilized with 0.5% Triton X-100 for 20 minutes at room temperature. Organoids were then blocked with 2% donkey serum for 1 hr and incubated with primary antibody overnight and washed in PBS containing 0.01% Triton-X 100. Secondary antibody incubation was also performed overnight in gastric organoids, and subsequently immunostained for cell nuclei using 10 μg/mL Hoechst. The following primary antibodies and dilutions were used: 1:100 human and mouse -specific rat anti PD-L1 (Novus Biologicals, NBP1-43262), 1:100 human and mouse-specific rabbit anti Gli-2 (Novus Biologicals, NBP2-23602), 1:100 human and mouse-specific rabbit anti PD-L1 (Novus Biologicals, NBP1-76769), 1:100 human-specific rabbit anti-Histone H1.0 (abcam, ab125027), 1:200 mouse alpha smooth muscle actin (SMA, Novus Biologicals, NBP2-22120) or 1:2000 dilution of goat anti-intrinsic factor (IF, a kind gift from Dr. David Alpers, Washington University School of Medicine in St. Louis). Both slides and whole mount organoids were imaged on a Zeiss LSM710 LIVE Duo Confocal Microscope.

### Immunohistochemistry

Stomach tissues were fixed in 4% paraformaldehyde for 15 minutes, paraffin embedded, and 5 μm sections were cut. After deparaffinization, antigen retrieval was performed by heating the slides for 10 minutes at 100°C in 0.01M sodium citrate buffer (Antigen Unmasking Solution, Vector Laboratories, Burlingame, CA). Endogenous peroxidase activity was then blocked by incubating slides in 0.3% hydrogen peroxide/methanol for 20 minutes followed by 20 minutes incubation with 20% goat serum. Slides were incubated with either 1:100 dilution of PCNA antibody (Novus Biologicals, NB100-456), PD-L1 antibody (Novus Biologicals, NBP1-76769) or Ki67 (Fisher scientific, Rm-9106RQ) overnight at 4°C. Slides were then incubated with biotinylated anti-rabbit IgG secondary antibody for 30 minutes followed by additional 30 minute incubation with ABC reagent (Vectastain ABC kit; Vector Laboratories, Burlingame, CA). The color was developed with 3,3′-diaminobenzidine (DAB) using the DAB Substrate Kit (Vector Laboratories, Burlingame, CA) and counterstained with hematoxylin (Fisher Scientific Company, 245–653), dehydrated and mounted with Permount (Fisher Scientific company, SP15-100).

Paraffin embeded stomach tissues were also labeled for PCNA and CD8 antigens using ImmPRESS reagents. Briefly, after the blocking of endogenous peroxidase activity, the slides were blocked with 2.5% normal horse serum. The slides were then incubated with 1:2000 dilution of PCNA antibody (Novus Biologicals, NB100-456) overnight at 4°C, followed by 30 minute incubation with anti-rabbit ImmPRESS Ig (Vector Lab, MP-7401) and color was developed using peroxidase substrate solution (Vector lab, SK-4605). After washing, slides were blocked for a second time with 2.5% normal goat serum, and incubated with 1:200 dilution of CD8 antibody (Novus Biologicals, NBP1-49045) overnight at 4°C, followed by 30 minute incubation with anti-rat ImmPRESS Ig (Vector Lab, MP-7444) and color developed using peroxidase substrate solution (Vector lab, SK-4105). After completion of immunohistochemical staining, slides were counterstained with Methyl Green (Vector lab, H-3402) and mounted with Permount (Fisher Scientific Company, SP15-100).

### Statistical analyses

The significance of the results was tested by one-way ANOVA or student's *t*- test using commercially available software (GraphPad Prism Software, San Diego, CA). A *P* value < 0.05 was considered as the level of significance.

## SUPPLEMENTARY MATERIALS FIGURES AND TABLES







## Abbreviations

PD-L1: Programmed cell death - ligand 1
PD-1: Programmed cell death protein - 1
PD-L1Ab: Cytotoxic T Lymphocytes
CTL: PD-L1 neutralizing antibody
CD8: Cluster of differentiation 8
Hh: Hedgehog
DC: Dendritic cells; *iLgr5*
*GLI2A*: triple-transgenic *Lgr5-EGFP-IRES-CreERT2; R26-LSL-rtTA-IRES-, EGFP; tetO-GLI2A* mouse
PBS: Phosphate buffered saline
DPBS: Dulbecco’sPhosphate buffered saline
qRT PCR: Quantitative reverse transcription polymerase chain reaction
mTGO: Mouse tumor derived gastric organoids
mGO: Mouse gastric organoids
huTGO: Human tumor derived gastric organoids
EDTA: HBSS: Hank's Balanced Salt Solution
DMEM: Dulbecco's Modified Eagle's medium
EGF: Epidermal growth factor
FGF10: Fibroblast growth factor 10
FCS: Fetal Calf Serum
rmGMCSF: Recombinant mouse granulocyte-macrophage colony stimulating factor
CM: Conditioned media
RPMI: Roswell park memorial institute medium
BSA: Bovine serum albumin
5FU: 5 Fluorouracil
PCNA: Proliferating cell nuclear antigen
IF: Intrinsic Factor.
