# Supplementary material for: Hedgehog signaling induces PD-L1 expression and tumor cell proliferation in gastric cancer
Source: Oncotarget. 2018 Dec 21;9(100):37439–57. doi: 10.18632/oncotarget.26473 (PMC6324774; doi:10.18632/oncotarget.26473)
Supplement: Supplementary file 1 [file oncotarget-09-37439-s001.pdf]

Hedgehog signaling induces PD-L1 expression and tumor cell proliferation in gastric cancer

SUPPLEMENTARY MATERIALS

Supplementary Video 1: Representative time-lapse video of mFGO/DC/CTL co-culture treated with PD-LI inhibitor.
See Supplementary_Video_1

Supplementary Video 2: Representative time-lapse video of mTGO/DC/CTL co-culture treated with PD-LI inhibitor.
See Supplementary_Video_2
